# Projected Increases in Precipitation Are Expected To Reduce Nitrogen Use Efficiency and Alter Optimal Fertilization Timings in Agriculture in the South East of England

**DOI:** 10.1021/acsestengg.1c00492

**Published:** 2022-06-09

**Authors:** Dan McKay Fletcher, Siul Ruiz, Katherine Williams, Chiara Petroselli, Nancy Walker, David Chadwick, Davey L. Jones, Tiina Roose

**Affiliations:** † Bioengineering Sciences Research Group, Department of Mechanical Engineering, School of Engineering, Faculty of Engineering and Physical Sciences, University of Southampton, Southampton SO17 1BJ, U.K.; ‡ School of Natural Science, Environment Centre Wales, Bangor University, Bangor, Gwynedd LL57 2UW, U.K.; § SoilsWest, UWA School of Agriculture and Environment, The University of Western Australia, Perth, WA 6009, Australia; ∥ Faculty of Science and Health, University of Portsmouth, Portsmouth PO1 2DT, U.K.; ⊥ Dipartimento di Chimica, Biologia e Biotecnologie, Università degli Studi di Perugia, Perugia 06125, Italy

**Keywords:** nitrogen use efficiency, precipitation, agriculture, modeling, climate change

## Abstract

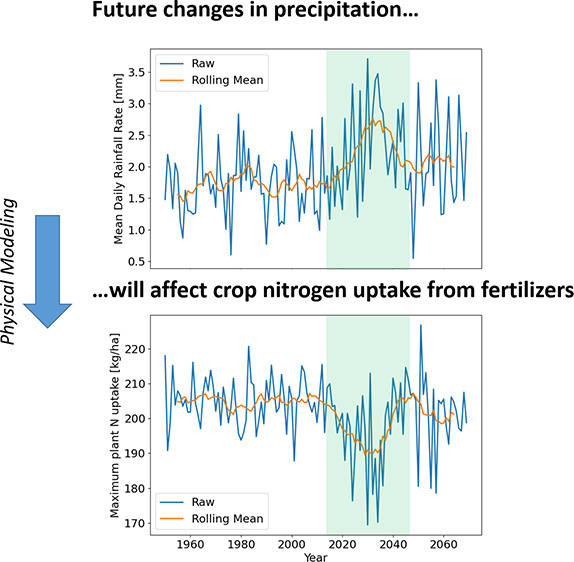

Nitrogen fertilization is vital for productive agriculture and efficient land use. However, globally, approximately 50% of the nitrogen applied is lost to the environment, causing inefficiencies, pollution, and greenhouse gas emissions. Rainfall and its effect on soil moisture are the major components controlling nitrogen losses in agriculture. Thus, changing rainfall patterns could accelerate nitrogen inefficiencies. We used a mechanistic modeling platform to determine how precipitation-optimal nitrogen fertilization timings and resulting crop nitrogen uptake have changed historically (1950–2020) and how they are predicted to change under the RCP8.5 climate scenario (2021–2069) in the South East of England. We found that historically, neither precipitation-optimal fertilization timings nor resulting plant uptake changed significantly. However, there were large year-to-year variations in both. In the 2030s, where it is projected to get wetter, precipitation-optimal fertilization timings are predicted to be later in the season and the resulting plant uptake noticeably lower. After 2040, the precipitation-optimal uptakes are projected to increase with earlier precipitation-optimal timings closer to historical values, corresponding to the projected mean daily rainfall rates decreasing to the historical values in these growing seasons. It seems that the interannual variation in precipitation-optimal uptake is projected to increase. Ultimately, projected changes in precipitation patterns will affect nitrogen uptake and precipitation-optimal fertilization timings. We argue that the use of bespoke fertilization timings in each year can help recuperate the reduced N uptake due to changing precipitation.

## 1 Introduction

Insufficient levels of available soil nitrogen (N) is a major limiting factor for crop yields globally.^1^ Soil replenishment of N occurs via a number of anthropogenic and natural processes.^2^ While biotic N fixation, i.e., converting atmospheric N to plant-available species, is one major pathway for soil N replenishment, synthesized N fertilizers via the Haber–Bosch process^3^ are necessary to support the current global food demand. Fifty percent of food production relies on synthesized fertilizers. However, their synthesis is energy-intensive, requiring 1.2% of global primary energy production.^4^

In addition to N fertilizer production, fertilizer application can also contribute to environmental issues. Transformations between N species can result in the release of potent greenhouse gases such as nitrous oxide (N_2_O).^5,6^ N added to fields can be flushed through the soil to deeper sections and/or into the water table (i.e., “leaching”), thus becoming inaccessible to the crops and causing eutrophication.^7,8^ Furthermore, N leached from fields into the groundwater has the potential to be denitrified into N_2_O in aquatic and marine environments.^9^ Additionally, ammonium in the soil can be volatized, and N can be released as ammonia gas; this can be significant (up to 60% of applied N) when the fertilizer is not incorporated into the soil and depends on temperature, soil texture, moisture, and pH.^10,11^

Soil moisture controls both N leaching and crop N uptake.^8,12−14^ High rainfall rates can flush N through the soil, resulting in increased leaching. However, low soil moisture limits N mobility, resulting in poorer plant N uptake.^11,15,16^ It remains unclear how precipitation patterns, soil type, crop, and growth stage influence uptake. However, it is clear that precipitation patterns are closely linked to nitrogen use efficiency (NUE)^17,18^ defined in this paper as the ratio of N taken up by the crop to the amount of N applied, i.e., NUE = (Quantity of plant assimilated N)/(Quantity of N input into the system).

Several studies have correlated cumulative rainfall with measures of N loss or plant N uptake.^13,17^ In field trials in England, Powlson et al.^16^ found that N loss correlated positively with total rainfall 3 weeks post fertilization, which explained 55% of the variation. This indicated that in this region, more rainfall results in lower NUE provided that water is not limiting for crop growth. In a mechanistic-modeling study, McKay Fletcher et al.^18^ found that cumulative rainfall post-fertilization explained 40% of the variation in N loss by only varying precipitation patterns between simulations (i.e., soil type, root growth, etc. were kept constant). The positive correlation between cumulative precipitation and N loses is only valid provided that there is enough water to support healthy crop development. In fact, in drier regions, NUE increases with cumulative precipitation, likely due to increased N mobility and enhanced crop growth, until a certain amount, from which it decreases due to enhanced leaching.^17^

Efforts to maximize N uptake focus on the Four Rs of fertilizer efficiency: “right source, right rate, right time, and right place”.^19^ However, strategies depend on the individual farms, meteorological condition, crop, and soil.^20^ “Right time” typically concerns timing the fertilizer application to ensure that N is available when the crop demand is the highest.^21^ Fertilization timing in agriculture is often based on the crop growth stage.^22,23^ Typical guidance for nutrient management in the United Kingdom can be found in Roques et al.^23^ Wallace et al.^24^ found that delaying fertilization until the end of tillering increased NUE except in very dry seasons where late fertilization decreased NUE. The physics-based model of McKay Fletcher et al.^18^ mirrored these results, finding that reduced N-uptake in drier seasons with late application was due to low N mobility. Delaying fertilizer application beyond the onset of stem elongation in wheat can also decrease yields,^25^ a feature that was also present in the model results.^18^ There are few studies that specifically investigate precipitation-optimal fertilizer timings, defined here as application timings that achieve maximum crop N uptake with respect to precipitation. Typically, fertilizer timings are based on crop growth stage in scientific experiments, the effect of rainfall is only mentioned to help explain anomalous results and not the primary control variable for fertilization timing (e.g., Dharmakeerthi et al.^26^ and references above). It is clear that better timing of N fertilization with respect to rainfall patterns (known as precipitation-optimal timings in the current study) can improve NUE in addition to timing with respect to crop demand.^24^ The former approach is the least studied but most volatile due to changing local climates, and hence both play an import role in plant N uptake.

The impact of climate change on N fertilization is becoming increasingly studied due to its sensitive dependence on weather.^13^ Changing weather, specifically heavy rainfall events, can increase N leaching and denitrification, resulting in increased N_2_O and N_2_ emission, lower crop NUE, and water pollution.^27^ In response, farmers need to adapt to ensure profitable production (i.e., enough crop N uptake) while minimizing adverse environmental impacts. Researchers have found moderate success with current approaches for mitigating N loss.^20^ Interviews with maize farmers in the mid-western United States revealed that they primarily responded to increased heavy rainfall events with increased fertilizer application.^28^ Although this maintains production, it also increases pollution. To enable sustainable N farming strategies, it will be necessary to demonstrate that strategies maintain high yields, lower pollution and incentivize farmers with reductions in net fertilizer costs.^28^ However, there are few studies that quantify the outcome of fertilization strategies in a changing climate or how optimal strategies may need to change.

Here, we studied precipitation-optimal N fertilization timings through a number of historic and predicted growing seasons in the South East of England using a mathematical model. We considered modeled crops of maize on a silt loam soil sown in spring. We used historic daily rainfall data from 1950–2020 and predicted daily rainfall data for 2021–2069 under the RCP8.5 climate scenario.^29^ Precipitation-optimal split fertilization timings (two fertilization days per growing season) were determined for each year by monitoring every possible fertilization day pair in the model and the resulting final modeled crop uptake. With this approach, we addressed the following questions for the South East of England climate scenario:

- Have precipitation-optimal fertilization timings and corresponding NUE changed historically?
- Are they projected to change?
- Do precipitation metrics correlate with precipitation-optimal fertilization times and/or NUE?

By answering these questions, we can inform how N fertilization strategies may be adapted and demonstrate the positive economic and environmental impact, in terms of NUE, of adapting to mitigate the effects of changing precipitation patterns. Finally, we argue that advanced computational tools can become valuable as support tools for farmer/agronomist decision.

## 2 Methods

### 2.1 Precipitation Data

We simulated a growing season from the 1st of March to the 30th of June using the precipitation data from the same period as an input to the model. Historic (1950–2020) daily precipitation data from the administrative region of South East of England were obtained from the Met Office using an average over weather stations in the region.^30^ Additionally, predicted daily precipitation data (2021–2069) for the same region under the RCP8.5 climate scenario were obtained from the UK Climate Projections User Interface (https://ukclimateprojections-ui.metoffice.gov.uk). The RCP8.5 climate scenario assumes a 3.2–5.4 °C increase in global mean surface temperatures averaged over years 2081–2100 compared to the preindustrial averages from years 1850–1900. The climate model used to predict the daily precipitation rates was HadGEM3-GC3.05 collected through the UK Climate Projections User Interface.^31^ The details of the configuration to access the data can be found in Williams et al.^32^

### 2.2 Precipitation Analysis

A number of precipitation metrics were used to infer how NUE and precipitation-optimal fertilization timings may correlate with precipitation patterns. Most simply, the mean daily precipitation rate for the growing season was calculated. When it was necessary to account for the large variations in precipitation from year-to-year and capture long time-scale changes, measurements and averages were taken over decades (interdecadal analysis). When referring to a specific year, we write it nonplural, e.g., 2020, and when referring to the decade, we write it plural, e.g., 2020s.

**Figure 1:**
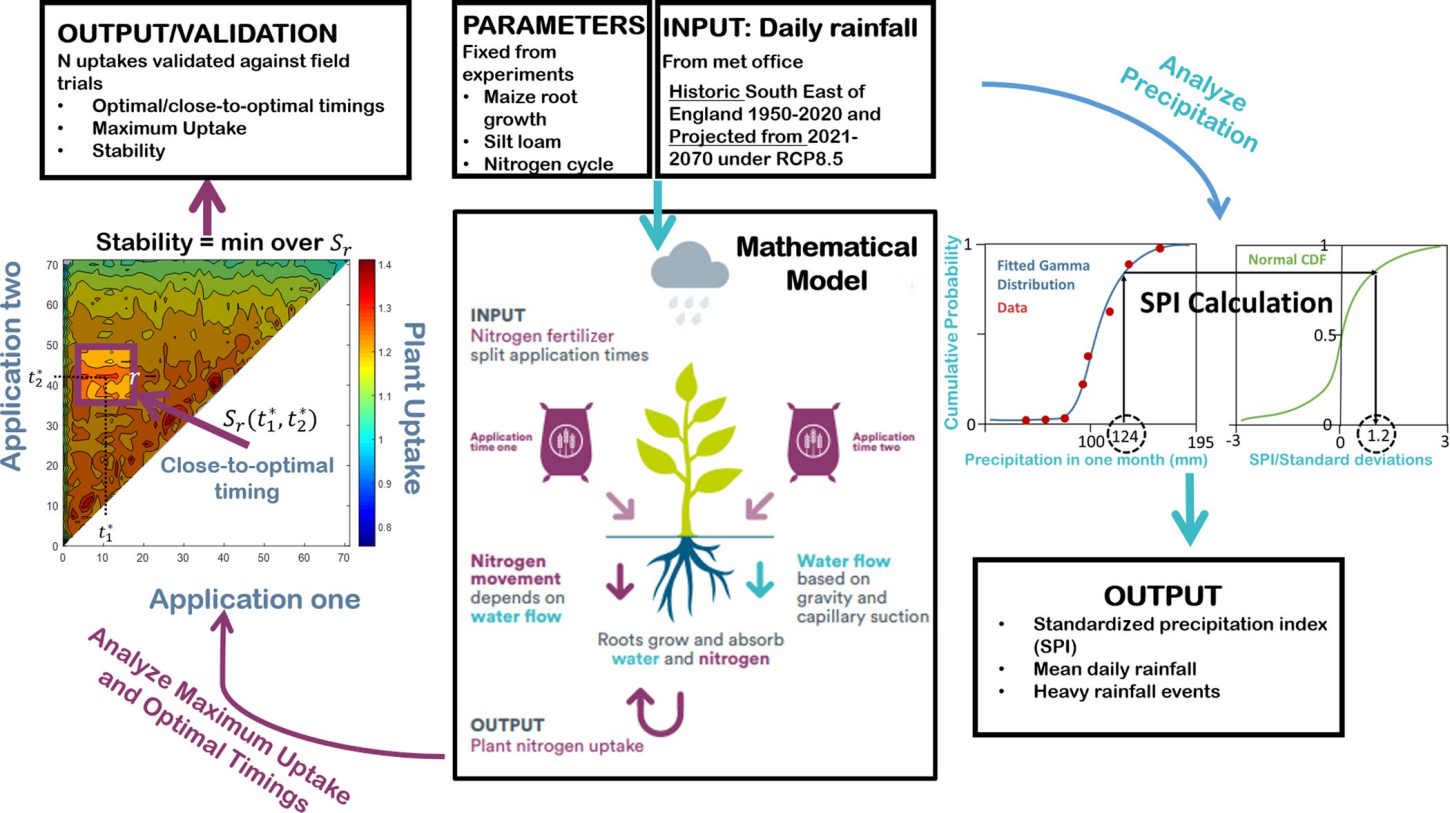
Explanation of modeling and processing of model outputs and rainfall data. Historic and projected rainfall data are an input to the mechanistic model. The model is solved for every possible split fertilization timing, and the results are analyzed, including “stability”, maximum uptake, and optimal fertilization timings. In addition, the rainfall data are analyzed using a one-month aggregated standardized precipitation index (SPI), heavy rainfall events, and means.

Precipitation variability is expected to increase, resulting in increased heavy rainfall events and droughts.^33^ In the context of N fertilization, a heavy rainfall event over 1 day or less can have a large impact on N leaching. To account for this, we define a “heavy rainfall event” as days with high rainfall rates relative to a reference period.^27^ The period 1950–1979 (March to June) is used as a reference period, and the daily rainfall rate, which marks the top one percentile in this reference period, is calculated. A heavy rainfall event is then defined as any day that is equal to or above this top one percentile rainfall rate.^27^ Since 1 day without any precipitation is common and has much less impact on soil moisture than a heavy rainfall event, defining lack of rainfall in the context of N fertilization requires a longer time scale. A common approach to measure drought is the Standardized Precipitation Index (SPI).^34^ The SPI measures standard deviations from the mean over aggregated time-periods, typically 1, 3, 6, 18, 24 months depending on the context in which drought is defined. To calculate the SPI, a probability density function (gamma distribution in this paper) is fitted to the aggregated rainfall data using the maximum-likelihood approach (find distribution-parameters in which the data are most probable when drawn from that distribution). The fitted cumulative density function is then calculated and transformed to standardized normal cumulative density function to determine the SPI as standard deviations from the mean; see the SPI calculation in Figure 1 for a visual description of this index. SPI measurements of drought are thus relative to the region. Since precipitation-optimal fertilization timings depend on changes in soil moisture, we chose the shortest viable time aggregation of 1 month for this study. Thus, four SPIs were given per growing season in the simulations. The classification of relative droughts using the SPI are as follows: 0 ≥ SPI > – 1 mild drought, −1 > SPI > – 1.50 moderate drought, −1.5 ≥ SPI > – 2 severe drought, and SPI ≤ – 2 extreme drought.^34^ For each decade, we calculate the percentage of months that are moderate drought and above or severe drought and above. The SPI was calculated in Python3 (Python Software Foundation, https://www.python.org/) using the standard_precip package (https://github.com/e-baumer/standard_precip).

### 2.3 Modeling

The modeling framework follows that of McKay Fletcher et al.^18^ Here, we summarize the approach and highlight important assumptions in the model that are required to interpret the results in the relevant context. We aim to simulate spring sown maize on a silt loam in the South East of England. Split fertilization timings will then be varied for each year from 1950 to 2059. The model couples the advection–diffusion-reaction equation for N transport and the N cycle in soil to Richards’ equation for water flow in soil. Importantly, the advective N transport is governed by the soil saturation profile to accurately capture the effect of soil moisture and precipitation on N dynamics. The crops are represented by a root length density function and a root depth function that evolves in time according to logistic root growth equations with parameters that match the growth of maize. The crops absorb the N species and water in soil. Growth stage-dependent crop N uptake is not explicitly considered in the model as our emphasis is on precipitation pattern variation. However, N demand is a function of root length density, which itself is a proxy for plant size. Thus, the growth stages happen at the same time each year. The model assumes there is the equivalent of 41.6kg N ha^−1^ of nitrate, 6.6 kg N ha^−1^ of ammonium and 191 kg N ha^−1^ of N in organic matter distributed throughout the soil depth initially before each simulation starts. Nitrate and ammonium are both immediately available to the plant, but N in organic form has to undergo reversible bio-mediated reactions into nitrate or ammonium to be available for plant uptake. Figure S1 shows the performance of the model against the experimental data of Powlson et al.^16^ by correlating N leaching with cumulative rainfall 3 weeks post fertilization. The model data in this figure used daily rainfall rates drawn from a distribution that was fit to rainfall data in the South East of England. We refer the reader to McKay Fletcher et al.^18^ for a full description of the model. It is important to note that the root depth and length density functions are independent of water and N uptake; i.e., plant growth is never water or N limited. This might become relevant when interpreting the results regarding the drier years where water may be limiting. However, the region of study, the South East of England, is a temperate region and is rarely water limited for grain production. Additionally, gaseous losses of N (e.g., N_2_O, N_2_, and NH_3_) from the system are not explicitly included in the current version of the model. Typically, only fractions of a percent of nitrate is transformed into nitrous oxide during denitrification in agriculture.^35^ Although we judged this to have little effect on crop N uptake and omitted it from the model for parsimony, nitrous oxide is a potent greenhouse gas and should be included in future models considering greenhouse gas emissions. Ammonia volatilization can contribute to a significant amount of N loss from soil systems; however, for ammonium nitrate, the fertilizer simulated in this study, losses are typically between 2 and 3% of the applied N, which we judged to be small enough compared to leaching to omit from the model.^11^ Therefore, N loses calculated by the model only include leaching and any link between N losses and NUE is an approximation.

The experimental (input) variables, namely, the precipitation pattern, and the two N fertilization applications are boundary conditions on the soil surface for Richards’ equation and the N advection–diffusion–reaction equation, respectively. The applications of N fertilizer are modeled as pulses of ammonium nitrate at user-controlled fertilization times *t*_1_ and *t*_2_. The fertilizer is applied at a yearly rate equivalent to 144 kg ha^–1^ (a typical recommendation for maize to maximize yield and reduce leaching^8^), with one third being applied at *t*_1_ and the remaining two thirds applied at *t*_2_. One instance of the model refers to a specific growing season’s precipitation pattern and a fertilization timing pair (*t*_1_, *t*_2_); from the solution of the model, the plant N uptake can be calculated by integrating the root uptake soil sink over space and time. The fertilization timings are limited to the first 70 days of the growing season with *t*_1_ ≤ *t*_2_ ≤ 70 days. For each growing season (i.e., precipitation pattern), the fertilization timing pair (*t*_1_, *t*_2_) that achieves the maximum crop N uptake is calculated directly. Specifically, the model is solved for every possible fertilization timing pair with 1.2 day resolution in fertilization timing, and the total N uptake is calculated. This results in data demonstrated in the heat map in the left of Figure 1 for each year. The fertilization timing pair that achieves the maximum plant N uptake relative to the growing season is referred to as the precipitation-optimal timing and the associated N uptake is referred to as the maximum uptake. Each model instance was solved numerically using a finite element method in Comsol 5.3a (COMSOL AB, Stockholm, Sweden).

### 2.4 Modeling Analysis

To determine the precipitation-optimal timing for all growing seasons, 111,600 instances of the model were solved numerically. As with the precipitation analysis, the results are presented in both yearly and decadal groupings to determine both short and long time-scale trends. The use of an exhaustive approach as opposed to an optimization method enabled the calculation of fertilization timing pairs in the growing season that achieve close-to-maximal N uptake relative to the growing season. A fertilization timing pair is said to be close-to-optimal if it achieves an N uptake within 5% of the precipitation-optimal timing in that growing season. A growing season with many close-to-optimal timings is advantageous as fertilization strategies can be less accurate and the farmer can choose when to fertilize based on other factors besides precipitation, e.g., growth stage.

It is possible that close-to-optimal timings follow or predate timings that achieve low N uptakes. Ideally, close-to-optimal timings are surrounded by fertilization timings that achieve relatively high uptakes so that the farmer has a buffer zone to fertilize in. We developed a metric to quantify this feature and determine how this has changed and is predicted to change: For a given close-to-optimal timing pair, (*t*_1_^*^, *t*_2_^*^) in a particular growing season, denote the set of all timings within radius *r* days each side of (*t*_1_^*^, *t*_2_^*^) by *S_r_*(*t*_1_^*^, *t*_2_^*^). The “stability” of (*t*_1_^*^, *t*_2_^*^) is defined as the minimum uptake achieve by the fertilization timing pairs in *S_r_*(*t*_1_^*^, *t*_2_^*^) as a proportion of the uptake achieved by fertilizing on (*t*_1_^*^, *t*_2_^*^), see Figure 1 (small box on heat map) for a visual description of stability. The “stability” of a growing season is then defined as the mean stability over all close-to-optimal timings in the growing season. For example, a growing season with a stability of 0.75 means that, on average, a farmer is guaranteed to get within 75% of the close-to-optimal timing if they miss the close-to-optimal timing by *r* days either side. We present analysis of stability with *r* = 2.4 days. To analyse trends in precipitation-optimal fertilisation timings and uptakes with respect to yearly mean daily rainfall rate and mean SPI we report the Pearson’s correlation coefficient. All analysis of the model results was computed in Python3.^36^

## 3 Results

### 3.1 Precipitation History and Projections

**Figure 2:**
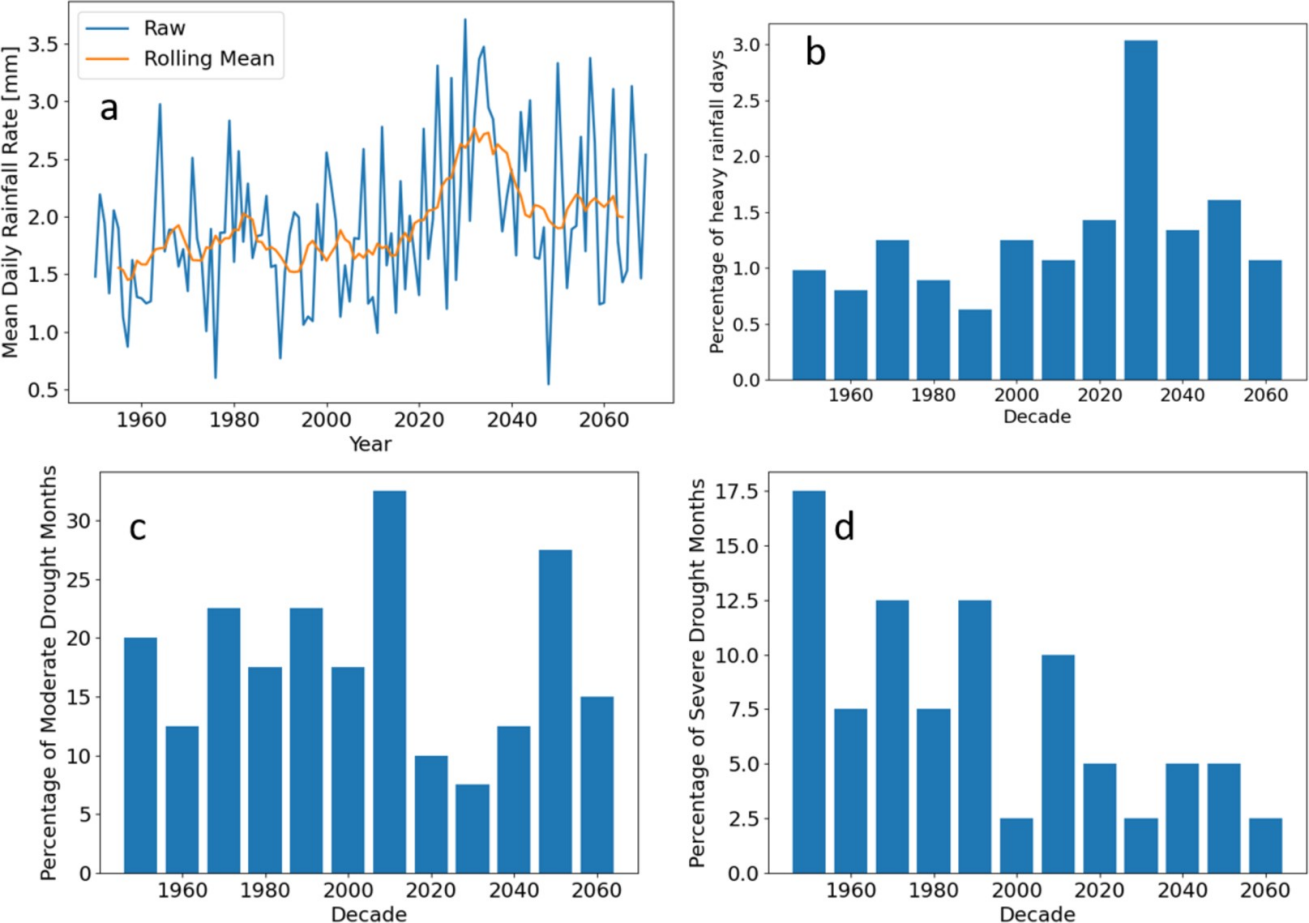
Analysis of precipitation data within the growing seasons. (a) Yearly mean March–June daily rainfall. The rolling mean with width 11 years is also shown. (b) Percentage of days classified as a heavy rainfall event in each decade. A heavy rainfall event is a day higher than the top percentile of daily rainfall rates from 1950 to 1979. (c) Percentage of months in the decade classified as moderate drought or worse, SPI ≤ −1.0. (d) Percentage of months in the decade classified as severe drought or worse, SPI ≤ −1.5.

We found a large interannual variability in the mean daily rainfall rate, Figure 2a. From 1950 to 2021, the rolling mean (width 11 years) hovered around 1.7 mm day^–1^. After 2021, the rolling mean is projected to monotonically increase until it reached a maximum in 2032, where the raw values are projected to reach 3.71 mm day^–1^. The rolling mean was then projected to decrease until 2045 and then hover around 1.9 mm day^–1^. From 1980s to 2010s, the heavy rainfall days stayed close to 1%, suggesting that there was little change from the reference years in this period, Figure 2b. In the 2030s, there was a steep jump to 3.1% of heavy rainfall days, after which the heavy rainfall events were projected to decrease back to the values of the 2020s. The number of moderate drought months from 1950s to 2020s stayed between 13 and 22%, Figure 2c. The 2020s, 2030s, and 2040s were projected to have noticeably lower amounts of moderate drought months, Figure 2c, which is unsurprising given the projected high daily rainfall rates, Figure 2a. This analysis suggests that the growing season had consistently drier months historically, while in the future, under this climate scenario, we expect these months to be interrupted by more heavy rainfall events.

### A Computational History and Projection of Nitrogen Uptake and Precipitation Optimal Fertilization Timings

#### 3.2.1 Nitrogen Uptake

**Figure 3:**
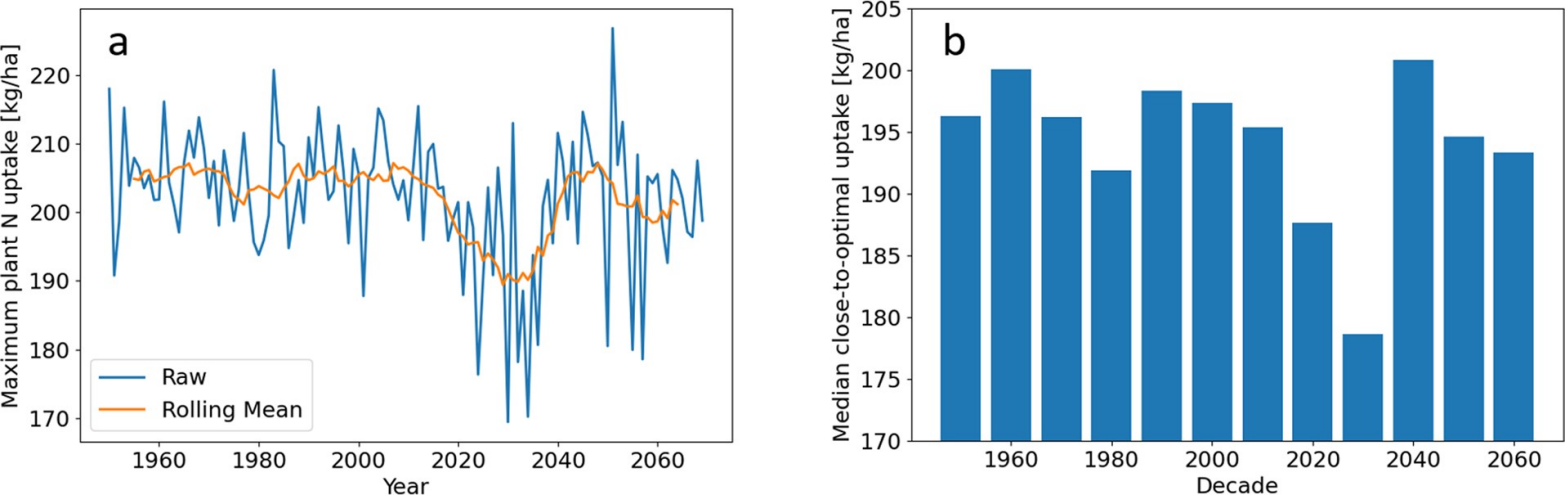
Modeled maximum nitrogen uptakes based on historical and projected climate data. (a) Maximum nitrogen uptake possible in each year from 1950 to 2069. The rolling mean with a window size of 11 years is also shown. (b) Median of all close-to-optimal uptakes in each decade. A close-to-optimal uptake is a plant nitrogen uptake within 5% of the maximum in its growing season.

The year on year maximum modeled N uptake is shown in Figure 3a. All “N uptake” results from this point onward are modeled values. For historic years (1950 to 2020), the model predicted the maximum N uptake to be around 204 kg N ha^–1^ (see the rolling mean in Figure 3a). However, there was large interannual variability. For example, in 1951, the maximum N uptake was 191.4 kg N ha^–1^. In the following year, this increased by 12% to 213.8 kg N ha^–1^. The rolling mean of N uptake started decreasing toward the end of the 2010s, where in 2030, it is predicted to reach a minimum of 190.0 kg N ha^–1^, with some specific years reaching lows of 169.1 kg N ha^–1^ (2030). This corresponds to increased mean projected rainfall and increased percentage of heavy rainfall events in the same period, Figure 2a,b. After 2034, the rolling mean is predicted to increase rapidly until 2043 to reach values similar to the historical maximum uptakes, which aligns with the mean projected rainfall rate decreasing in this period, Figure 2a. However, from 2053 to 2069, the rolling means of maximum N uptakes are predicted to fall below those of the historical data. In the projected years, the interannual variability in maximum uptake can be larger than the historical variability. For example, in 2030, the maximum uptake was 169.1 kg N ha^–1^, which increases by 25.6% to 212.4 kg N ha^–1^ in 2031. The maximum uptake over all of the years is predicted to be in 2051, achieving 226.35 kg ha^–1^. The model-predicted crop N uptakes are consistent with field trial measurements for maize. Ciampitti and Vyn^37^ found that mean N uptake for maize over a number of varieties and fertilization quantities was 152 kg N ha^–1^ with a maximum and minimum of 387 and 33 kg N ha^–1^, respectively. Our model predicted that mean N uptake over all fertilization timings ranged from 158 to 163 kg N ha^–1^, Figure S2.

Figure 3b illustrates a decadal analysis and considers the median over all close-to-optimal uptakes in each decade. This approach monitored and predicted longer time-scale changes. Additionally, median values over close-to-optimal (N uptakes within 5% of the maximum) values are reported to account for the fact that the true maximum is unlikely to be achieved in practice. Historically, there were only small changes from decade to decade. However, in the projected wetter decades of 2020s and 2030s, the median close-to-optimal uptake is predicted to drop dramatically before reaching the historical values again in the 2040s–2060s.

#### 3.2.2 Fertilization Timings

**Figure 4:**
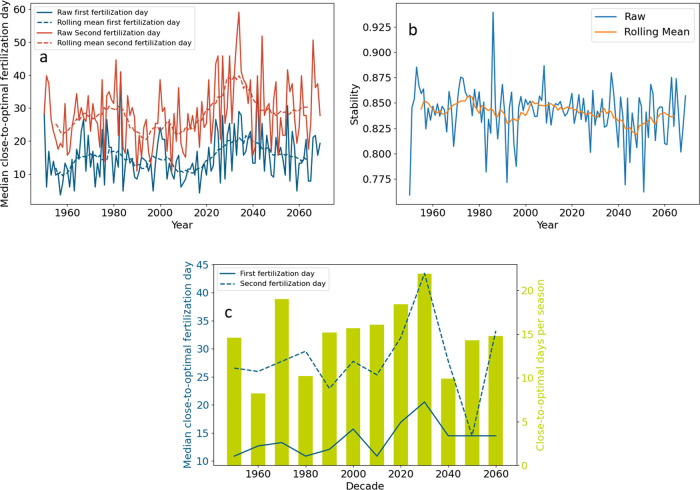
History and projection of precipitation-optimal fertilization timings and their stability. (a) Yearly analysis of the median close-to-optimal first and second fertilization timings. The rolling mean with a window size of 11 years is also shown. (b) Yearly stability with a 2.4 day window. Note, a growing season with a stability of 0.75 means that, on average, a farmer will get within 75% of the close-to-optimal timing if they miss the close-to-optimal timing by 2.4 days either side. The rolling mean with a window size of 11 years is also shown. (c) Decadal analysis of median precipitation-optimal fertilization days and number of close-to-optimal fertilization day pairs per season. A close-to-optimal fertilization day-pair is defined as those fertilization day pairs that achieve a nitrogen uptake within 5% of the maximum of that growing season. The median close-to-optimal first and second fertilization days and close-to-optimal uptake are taken over all close-to-optimal fertilization day pairs in that decade or year.

The median close-to-optimal first and second fertilization timings year-on-year can be seen in Figure 4a. As with the maximum N uptakes, there was large interannual variability both in the historic and the projected years. For example, in 1982, the precipitation-optimal first fertilization day was 12 days after germination, while in 1983, it was day 35. Additionally, there seemed to be more interannual variability in the second fertilization day than the first, which could be explained by the fact that twice as much fertilizer was applied in the second day. The rolling means of the two fertilizer application timings were positively correlated (Pearson’s correlation coefficient = 0.86); e.g., when one was later, the other was also later. In general, the same was true for the raw data, but the correlation was not as strong (Pearson’s correlation coefficient = 0.66), showing that different alterations in fertilization timings were required for each application during certain years. From 2015, the rolling mean for both timings is predicted to be increasingly later until 2030. For the first application, the rolling mean was predicted to be the latest around 2030, but the raw values are not predicted to exceed the historic values. After 2030, the rolling mean for both timings is predicted to become earlier and comparable to historic values. This corresponds with projected high rainfall followed by low rainfall in the same period, Figure 2a.

There was little change in stability year-on-year (see the rolling mean in Figure 4b). Stability can vary, with some years being as low as 0.76 and some as high as 0.94; however, this feature of precipitation-optimal fertilization timings did not, nor is it expected to, change significantly.

Decadal analysis for precipitation-optimal fertilization timings shows that, based on projected rainfall, by the 2030s, the timings will be significantly later than the historic timings, with the median optimal second application predicted to be at day 43 compared to around day 26 historically; see Figure 4c. Figure 4c also displays the number of close-to-optimal fertilization day pairs per growing season in each decade, which varies decade to decade. The 1960s only had 8 close-to-optimal fertilization day pairs per growing season, while the 2030s (the wettest decade according to projections) had 22. Ideally, there would be many close-to-optimal fertilization day pairs per growing season, so the farmer has many chances to time their fertilization successfully. Although the 2030s are predicted to have the most close-to-optimal fertilization day pairs per growing season, the 2030s also had the lowest max uptake, 178.9 kg N ha^–1^, Figure 3b. This means that the 2030s is predicted to have many chances to achieve a low maximum uptake relative to other decades.

### 3.3 Precipitation Metrics versus Maximum Nitrogen Uptake and Precipitation-Optimal Fertilization Timings

**Figure 5:**
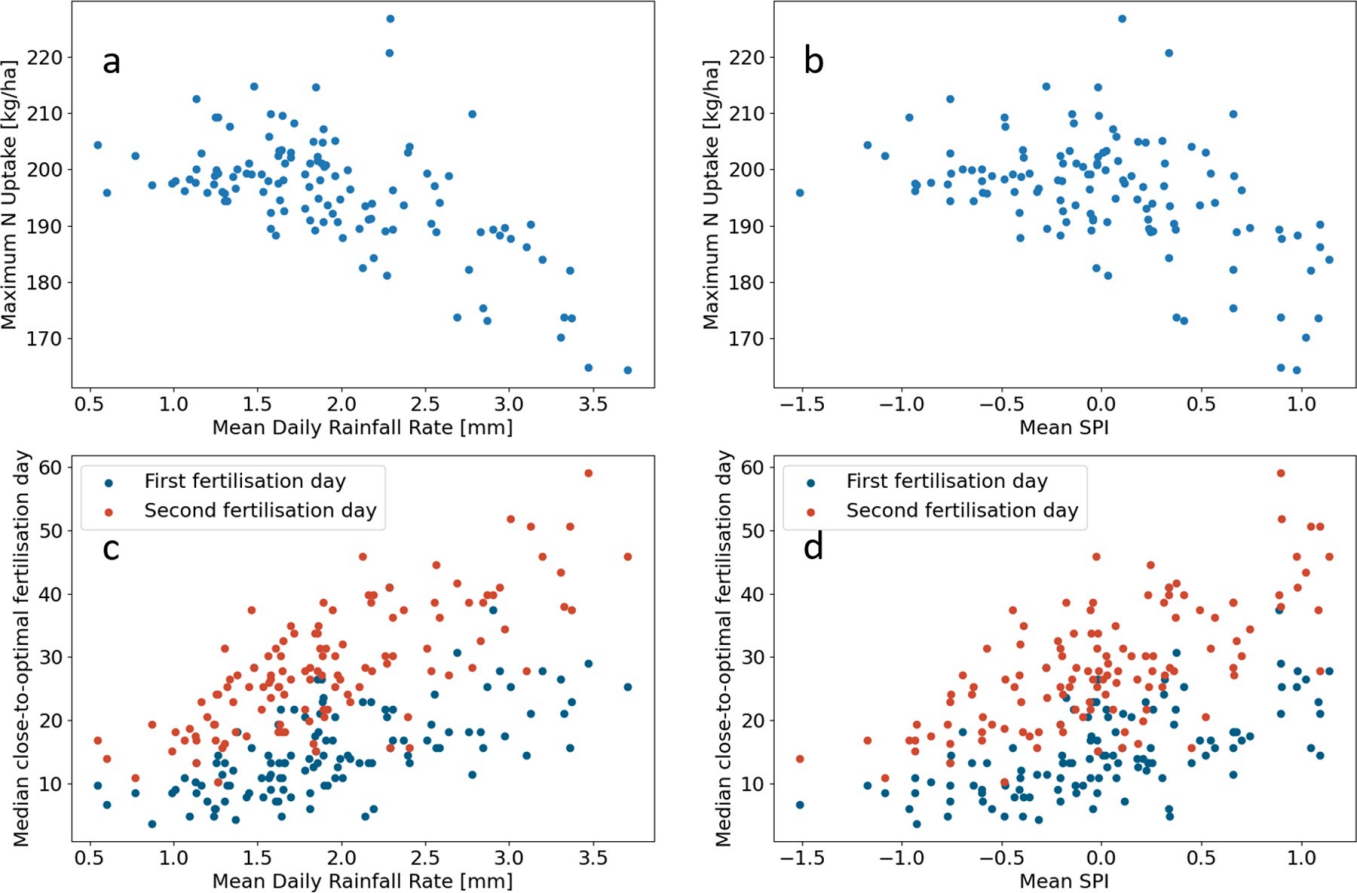
Correlations of yearly precipitation metrics with maximum nitrogen (N) uptake and precipitation-optimal fertilization days. (a) Maximum N uptake vs mean daily rainfall rate in the growing season (March–June); each dot is an individual year. (b) Maximum N uptake vs mean standardized precipitation index (SPI) in the growing season. The 1-month aggregated SPI is calculated for each of the 4 months in the growing season, and the mean is taken for each year. (c) Median close-to-optimal first and second fertilization day vs mean daily rainfall rate. A fertilization day is close-to-optimal if it achieves an N uptake within 5% of the maximum in that year. (d) Median close-to-optimal first and second fertilization day vs mean SPI.

Since projected precipitation patterns were speculative, correlations between precipitation metrics and maximum N uptakes or precipitation-optimal fertilization timings can help guide fertilization strategies in an uncertain future climate. We found that the mean daily rainfall rate correlated negatively with maximum N uptake with a Pearson’s correlation coefficient of −0.59, Figure 5a. Mean daily rainfall rates between 1.15 and 2.35 mm day^–1^ could achieve the highest maximum N uptakes, although rates above 2.15 mm day^–1^ could also result in low maximum N uptakes. Mean daily rainfall rates above 2.85 mm day^–1^ always had low maximum N uptake. The mean (one month aggregated) SPI of the growing season had less correlation with maximum N uptake than mean daily rainfall rates with a Pearson’s correlation coefficient of −0.46, Figure 5b. However, a mean SPI above 0.75 consistently resulted in low uptakes, while a mean SPI between −0.75 and 0.65 could result in high uptakes. Mean daily rainfall rates correlated positively with both the first and second precipitation-optimal fertilization timings, Figure 5c. The precipitation-optimal second application timing had a higher Pearson's correlation coefficient (0.75) than the first fertilization timing (0.62) with mean daily rainfall rate. This is because the second application contained twice as much fertilizer as the first, suggesting that the greater amount of fertilizer applied the greater dependence of precipitation-optimal timing on precipitation. Similar to the maximum N uptake, mean SPI showed a similar trend, but the correlation was less strong than mean daily rainfall rates for the first (Pearson’s correlation coefficient 0.59) and second (Pearson’s correlation coefficient 0.68) precipitation-optimal fertilization timings, Figure 5d.

## 4 Discussion

Recently, the dependence of N leaching on soil moisture/precipitation has been in the spotlight due to changing local precipitation patterns.^13,28,38^ Researchers have pointed out the importance of demonstrating both the environmental and economic benefits of adapting fertilization strategies to changing precipitation patterns.^28^ However, to our knowledge, there have been no attempts to directly quantify how changing precipitation patterns might affect crop N uptake or how fertilization strategies may need to change in the future to ensure high NUE in arable farming. Here, we used a well-established mechanistic physical modeling approach^39,40^ to study the effect of precipitation patterns on precipitation-optimal split fertilization timings to maximize plant N uptake. Importantly, N dynamics were coupled to water movement in the soil, so the effect of precipitation could be studied directly. As a case study, we modeled maize grown in spring on silt loam in the South East of England; thus, our results would likely change given a different soil texture or crop type. By using historic and projected (RCP 8.5) precipitation data in the model, we could determine how the precipitation-optimal timings and maximum uptakes have changed and might change in the future for these conditions.

Historically, the mean daily rainfall in the South East of England had little change in the rolling mean. There was, however, large interannual variability, which was more pronounced for projected years. From 2021, the rainfall is projected to increase until reaching a peak in 2030, Figure 2a. This was projected to be accompanied by more heavy rainfall events and less severe droughts, Figure 2. These predictions are in agreement with previous studies regarding precipitation in temperate regions such as the South East of England. A warmer climate will accelerate the global water cycle, which is thought to increase extreme precipitation events, i.e., more heavy rainfall events, but less rainy days.^41^ However, this is not the case for regions in the subtropics where precipitation is expected to decrease due to climate change.^42^ Thus, our results are only relevant to the region reported, and future studies should consider other climates with contrasting predicted future precipitation patterns. To apply the same approach to drier regions, where climate change is expected to have a big impact on NUE and water use efficiency,^43^ it would be important to include additional mechanisms in the model. In particular, the root growth model should be extended to include water and nitrogen limited growth. The assumption of water and nitrogen-independent growth was valid for arable fields in the South East of England where crops are rarely water or nitrogen deficient. However, in drier regions, crops may produce less biomass due to water deficiency and therefore have lower N demand, which will affect N uptake and leaching. In the drier cases, it would be important to control fertilization amounts as well as timing to account for the possibility of low biomass.^43,44^ Additionally, water scarcity would affect the nitrogen cycle in the soil and soil saturation-dependent reaction rates may need to be included to accurately capture this.^45^

Only one realization of the climate model was used in the simulations. However, the behavior of the climate realization used in this study was representative of the ensemble average of multiple climate realizations, but the particular variability may not be exactly representative of all possible future trends. Our approach still provides a more realistic example of fluctuations in rainfall patterns that could be expected and how these fluctuations will impact N acquisition by crops in these conditions. We also note that the RCP 8.5 climate scenario (business as usual) is hopefully not the guaranteed scenario. However, this is expected to be the scenario that most perturbs trends that follow from the historic data set. This scenario is also currently serving as the basis for global policies.^29^ As such, the selection of the RCP 8.5 projection is likely to be a useful representation of the projected precipitation trends used in this study.

The historic interannual variability in N uptake increased in the projected years, Figure 3a. However, only the wettest decade of the 2030s was projected to have notably lower maximum N uptake on the decadal scale (Figure 3b). This result has severe implications for NUE, as crop yields in this period are expected to be poor under the current application strategy. Historically, practitioners have compensated for this by applying more fertilizer in response to reduction in crop yields.^28,38^ While this might be a necessary strategy to sustain production for this decade, there will likely be enhanced N leaching and increased N_2_O emissions in this period. Furthermore, our predictions suggest that maintaining a compensatory strategy past this decadal dip would be suboptimal, as precipitation rates are expected to reduce back to their pre 2030s trends. As such, our model results can help inform strategies for insuring practitioners during suboptimal times.

Both precipitation-optimal fertilization timings were predicted to become noticeably later in the 2030s, Figure 4c. In addition, there were predicted to be more close-to-optimal fertilization day pairs in the 2030s, Figure 4c. It seems that if the weather is wetter, maximum N uptake is reduced, precipitation-optimal fertilization timings become later, and the number of close-to-optimal fertilization day pairs per growing season increases, Figure 4. However, this only means that there are predicted to be more days to achieve this lower maximum, Figure 3. This is confirmed by correlating precipitation metrics with precipitation-optimal timings and maximum N uptakes and is true for many wet growing seasons, Figure 5, not just those in the 2030s. This is attributed to the wetter years having increased chance of leaching.^46^ Thus, fertilizing later gives the roots as long as possible to establish before fertilizer application to intercept N.^18^ However, applying fertilizer too late means that there is less time in the growing season for the crop to take up and utilize the applied N.^18,24^ The precipitation-optimal timings for wet years find the balance between mitigating leaching and ensuring enough time for crop uptake. The driest years did not have the highest maximum N uptakes, Figure 5a but were higher than the wettest years. This is attributed to low mobility of N with low soil moisture limiting crop uptake.^18^ To account for the low mobility, the precipitation-optimal fertilization timings in dry years are predicted to be earlier than wetter years Figure 5c; in these years, there was predicted to be less risk of leaching. However, the model did not account for reduced root growth in very dry conditions; thus, the maximum uptake for the driest years (if they were water limited) may be an over estimate.

The current model assumes constant temperature and does not account for the effect of global warming in order to carefully study the effect of changing precipitation, a scenario relevant to South East England. However, changing temperature would alter important processes in the model, including evaporation, root growth^47^ and transpiration, and N transformation rates in soil,^38^ which may ultimately affect the results. Including these processes would introduce many additional unknown parameters and further uncertainty to the model. Furthermore, changing precipitation is thought to have a larger impact than temperature on controlling crop N uptake in temperate regions,^13^ which was why precipitation was the initial study for our model.^14^ However, temperature can strongly affect gaseous N loses. Ammonia volatilization increased threefold when the temperature increased from 25 to 45 °C in a lab experiment.^48^ Thus, future models should certainly consider gaseous N losses when modeling the effect of warming on crop N uptake. However, temperature increases are unlikely to be this extreme in the South East of England. Temperature and precipitation act in tandem to affect cropping systems, and both need to be studied to fully understand the impact of climate change on NUE. The model assumptions regarding temperature should be reconsidered in future modeling studies to refine the current predictions, expand them to include a wider geographical area, and have holistic understanding of the effect of climate change on worldwide crop N uptake.

Mean daily rainfall rates had a stronger correlation with maximum N uptakes and precipitation-optimal fertilization timings than the mean 1-month aggregated SPI, Figure 5. This suggests that N fertilization is more sensitive to short time-scale variations in precipitation. SPI is judged to be a poor indicator of N uptake compared to mean daily rainfall rates. While SPI provides a more intuitive presentation of precipitation patterns (i.e., relative drought and flood), it obscures the detail required to capture precipitation-optimal fertilization. Additionally, since the calculation of SPI requires fitting a distribution to the local precipitation data, the correlations may not generalize to other regions. The full detail in the rainfall pattern was used directly as a boundary condition for the model output and, although more complicated, may be required to predict NUE accurately.

Our analysis assumes that farmers find precipitation-optimal or close-to-optimal fertilization day pairs for each growing season. In fact, most timings achieve poor N uptakes in each decade (Figure S2), and finding the timings that achieve high uptakes is not a trivial task. If in the future farmers decided to use the mean precipitation-optimal timings based on historic data, on average they would achieve 87.7% of the potential maximum uptake in the projected years (but the potential maxima are projected to be lower in the future). By comparison, the same strategy in the historic years would achieve 89.3% of the potential maximum on average. Thus, not only are the precipitation-optimal N uptakes projected to decrease due to increased precipitation in the future, but timing fertilizations based on the status-quo will further increase N losses. There is little an individual farmer can do to directly stop climate change, but by adapting N fertilization timings for each year based on crop growth stage^23^ and precipitation they could recuperate some of the reduced N uptake caused by changing precipitation. This adaptation would also reduce the quantity of N fertilizer required to produce high yields, as well as reducing leaching and greenhouse gas emissions, which would help mitigate the climate impact of agriculture. Currently, there is no decision support tool available to guide farmers on when to fertilize based on forecasted weather. Ideally, field trial data would be used to create such a tool, but the model data presented in this paper provide the starting point to create tools that can use the past and forecasted weather to guide farmers with a good time to fertilize.^49^

To conclude, simulation results show that there has been little change in crop N uptake or precipitation-optimal fertilization timings historically. However, there has been notable variation year-to-year. In the 2030s, simulations project N uptake to reduce and precipitation-optimal timings to become later in the season in response to wetter weather and, in particular, increased occurrence of heavy rainfall events. In addition, the year-to-year variation in crop N uptake increases due to climate change. Fertilization strategies should stay flexible since simulations project optimal-fertilization timings to become earlier and N uptake to reduce in the 2040s to figures similar to the historic in response to a reduction in precipitation.

## Supporting Information Available

Information on model-data comparison and further uptake rates presented for decadal analysis; (Figure S1) performance of the model against the field trials in terms of nitrogen loss; model data from simulations using daily rainfall rates drawn from a distribution that was fit to the South East of England; relationship between rainfall in the 3 weeks following application of nitrogen and percentage of nitrogen lost; and (Figure S2) distribution of all possible uptakes in each decade included in this study (PDF)
