# Supplementary material for: Projected Increases in Precipitation Are Expected To Reduce Nitrogen Use Efficiency and Alter Optimal Fertilization Timings in Agriculture in the South East of England
Source: ACS ES T Eng. 2022 Jun 9;2(8):1414–24. doi: 10.1021/acsestengg.1c00492 (PMC9379908; doi:10.1021/acsestengg.1c00492)
Supplement: Supplementary file 1 — ee1c00492_si_001.pdf [file ee1c00492_si_001.pdf]

Supplementary Information for: Projected increases in precipitation are expected to reduce nitrogen use efficiency and alter optimal fertilisation timings in agriculture in the south east of England

D. McKay Fletcher[a], S. A. Ruiz[a], K. Williams[a,d], C. Petroselli[a,e], N. Walker[a], D. Chadwick[b], D.L. Jones[b,c], T. Roose[a,$]

[a]*Bioengineering Sciences Research Group, Department of Mechanical Engineering, School of Engineering, Faculty of Engineering and Physical Sciences, University of Southampton, SO17 1BJ, UK*

[b]*School of Natural Science, Environment Centre Wales, Bangor University, Bangor, Gwynedd, LL57 2UW, UK*

[c]*SoilsWest, UWA School of Agriculture and Environment, The University of Western Australia, Perth, WA 6009, Australia*

[d]*University of Portsmouth, Faculty of Science and Health, Portsmouth, PO1 2DT, UK*

[e]*Dipartimento di Chimica, Biologia e Biotecnologie, Università degli Studi di Perugia, 06125, Italy*

[$]corresponding author: T.Roose@soton.ac.uk, Bioengineering Sciences Research Group, Department of Mechanical Engineering, School of Engineering, Faculty of Engineering and Physical Sciences, University of Southampton, University Road, Southampton SO17 1BJ, UK

1 SUPPLEMENTARY FIGURES

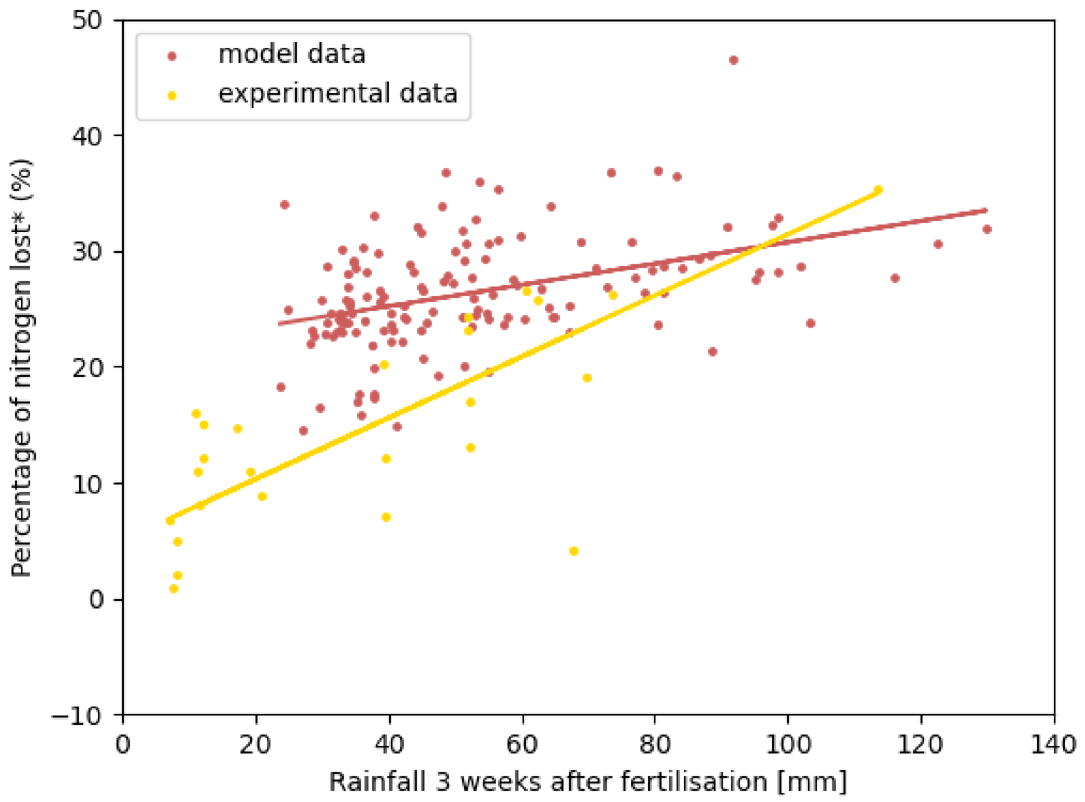

Figure S1: Verification figure redrawn from McKay Fletcher, et al. [1] demonstrating the performance of the model against the field trials of Powlson, et al. [2] in terms of nitrogen loss. The model data are from simulations using daily rainfall rates drawn from a distribution which was fit to the South East of England. The relationship between rainfall in the 3 weeks following application of nitrogen and percentage of nitrogen lost. In the model data (blue) 'loss' was defined as all nitrogen, both fertiliser and that originally in the soil, not recovered by the crop. In the model, both fertilisations were at day 5. The best fit line has a coefficient of determination $r^2 = 0.4$. The experimental data is from a field study in central England and redrawn from Powlson, Hart, Poulton, Johnston and Jenkinson [2]. In the experimental data (red) 'loss' was defined as isotopically labelled nitrogen applied as fertiliser (this does not include that originally in the s oil) which was not recovered in the plant nor in the first 70 cm depth of soil after harvest. The best fine line has a coefficient of determination $r^2 = 0.73$. This fit excluded the outlier point highlighted with the dashed circle. If this point was included, the resulting fit produced a coefficient of determination $r^2 = 0.55$

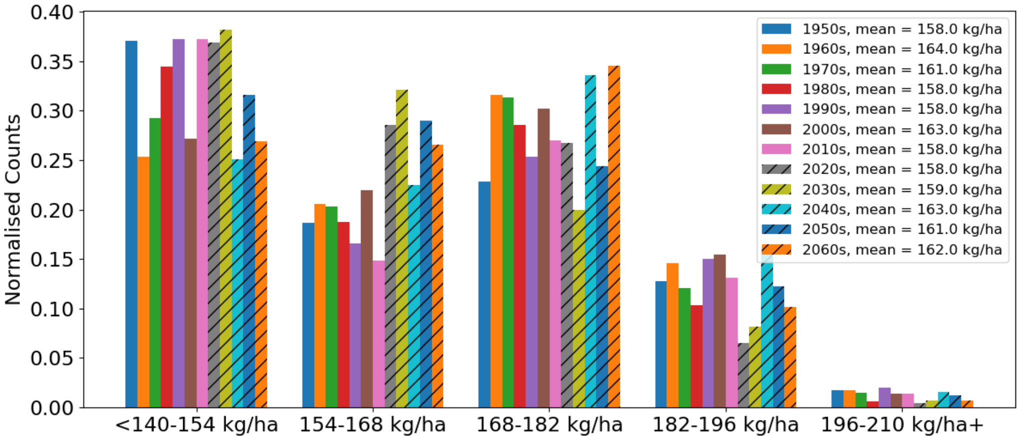

Figure S2: Distribution of all possible uptakes in each decade. The histogram is normalised so that each decades bins sums to 1. The projected decades are hatched and the historic decades are blocked. The means are also shown in the Legend.

1. McKay Fletcher, D.; Ruiz, S.; Dias, T.; Chadwick, D.; Jones, D.; Roose, T., Precipitation-optimised targeting of nitrogen fertilisers in a model maize cropping system. *Science of The Total Environment* **2021,** *756*, 144051.

2. Powlson, D.; Hart, P.; Poulton, P.; Johnston, A.; Jenkinson, D., Influence of soil type, crop management and weather on the recovery of 15 N-labelled fertilizer applied to winter wheat in spring. *The Journal of Agricultural Science* **1992,** *118*, (1), 83-100.
